# A Novel Encoded Excitation Scheme in a Phased Array for The Improving Data Acquisition Rate

**DOI:** 10.3390/s140100549

**Published:** 2013-12-31

**Authors:** César Gutiérrez-Fernández, Ana Jiménez, Carlos Julián Martín-Arguedas, Jesús Ureña, Álvaro Hernández

**Affiliations:** Electronics Department, University of Alcalá, Campus Universitario. Ctra. Madrid-Barcelona Km. 33.600, Madrid 28805, Spain; E-Mails: cesar.gutierrez@depeca.uah.es (C.G.-F.); cj.martin@depeca.uah.es (CJ.M.-A.); urena@depeca.uah.es (J.U.); alvaro@depeca.uah.es (A.H.)

**Keywords:** ultrasound imaging, phased arrays, CDMA (Code Division for Multiple Access), acquisition rate

## Abstract

One of the challenges of phased array (PA) ultrasonic imaging systems is their limited capability to deal with real-time applications, such as echocardiography and obstetrics. In its most basic outline, these systems require emitting and receiving with the entire array for each image line to be acquired; therefore, with many image lines, a higher acquisition time and a lower frame rate. This constraint requires one to find alternatives to reduce the total number of emissions needed to obtain the whole image. In this work, we propose a new PA scheme based on the Code Division Multiple Access (CDMA) technique, where a different code is assigned to each steering direction, allowing the array to emit in several directions simultaneously. However, the use of encoding techniques produces a reduction of the image contrast because of the interferences between codes. To solve this, a new scheme based on merging several images is proposed, allowing the system to get close to the theoretical maximum frame rate, as well as to limit the loss of contrast, intrinsic to the technique.

## 1. Introduction

Ultrasonic imaging systems based on phased array (PA) techniques have been widely used in medical diagnosis and industrial applications. These techniques have emerged as a safer and cheaper alternative to magnetic resonance and computed tomography, obtaining high quality images for clinical diagnoses. In industrial applications, PA techniques are also used for non-destructive tests (NDT), such as thickness inspection and defect detection in parts.

In PA ultrasonic technology, the beams generated with the array can be focused and steered, properly delaying the elements during the emission and reception of the signals. The basic idea is to define different lenses electronically, focusing the emitted energy on the desired region. PA techniques requires emitting and receiving with the entire array to obtain a line of the image. The more lateral of a resolution that is desired, the more lines that have to be acquired. Therefore, due to the limitation of the speed of sound, the acquisition rate decreases as the number of lines increases. This constraint is critical in some medical image applications, where it is necessary to scan organs, like the heart, that move quickly; and it becomes even more important in real-time 2D systems, where the emissions needed to acquire the data grow exponentially [1]. Therefore, it is necessary to find alternatives to reduce the number of emissions needed to obtain the image. In this work, an emission scheme based on encoded signals is proposed to achieve this goal.

To date, coded excitation and pulse compression have been already applied in PA imaging systems, mainly to enlarge the penetration depth and to improve the SNR (signal-noise ratio), without increasing the voltage driving levels. Most of these works use linear FM (frequency-modulated) signals. In [2], O'Donnell proposes a pseudo-chirp excitation, increasing the SNR by about 15 dB. In [3], Toosi presents a linear FM excitation based on chirp and minimum-variance adaptive beamforming to suppress the sidelobe levels, thus obtaining an SNR improvement of 18 dB.

Other works have also studied the application of encoding through binary codes. Moo-Ho in [4] proposes Golay codes to obtain SNR improvement without reducing frame rate. In [5], Kim presents a linear array emitting Golay codes in combination with chirp, achieving simultaneous multi-zone focusing along several scan lines.

Coded signals can be also used to discriminate simultaneous emissions. These techniques are known as Code Division Multiple Access (CDMA). CDMA techniques have been successfully used in indoor positioning and obstacle detection in robotics applications [6,7]. In these works, a multi-user scenario is considered, assigning a different code to every user. Then, simultaneous emissions and receptions from different users are possible, since each code is used as a user identifier. In that sense, CDMA techniques in imaging can provide a method to acquire data simultaneously, by scanning several lines at the same time, rather than the sequential one offered by conventional PA.

CDMA techniques have been already explored in PA systems. In [8], Shen proposes emitting a pseudo-orthogonal m-sequence in each direction, and using a bank of filters in reception of a parallel acquisition of several lines at the same time is achieved. Furthermore, Diego in [9] proposes a design of an ultrasonic array with M emitters and a single receiver based on encoded excitation with Kasami sequences for an obstacle detection application, which scans the whole environment with a single emission.

In this work, we propose an encoded excitation scheme with Kasami sequences in PA systems that allow parallel acquisition of the scan lines in order to increase the acquisition rate of the image. Thus, a different code is assigned to every steering direction to be explored, emitting all of them simultaneously. In reception, thanks to the orthogonality among codes, data coming from each direction can be discriminated after a correlation process. Hence, the theoretical maximum acquisition rate can be reached, scanning all lines of the image in only one emission.

Nevertheless, encoding techniques involve a loss of image contrast, mainly due to their inherent interference values. Therefore, another approach based on merging different images is also presented, allowing the system to get a frame rate reduction combined with suitable contrast.

This paper is organized as follows. Section 2 presents the pseudo-random sequences used in the encoded proposal. In Section 2.1, the basic outline of the proposal is described. An alternative to the basic scheme is presented in Section 2.2. System characterization is discussed in Section 2.3. In Section 3, simulation results for the encoded excitation scheme in comparison with conventional PA techniques are presented. Finally, some conclusions are discussed in Section 4.

## 2. Proposed Signal Processing

In the proposed system, by combining PA and CDMA techniques, the elements of the array are driven by encoded signals. The effectiveness of multiple-access techniques depends on the orthogonality features of the codes. These codes should have a suitable auto-correlation (AC) function with a high main lobe, in order to distinguish them from AC interference values, also called inter-symbol interference (ISI). Moreover, the codes should present low cross-correlation (CC) values to avoid multiple access interference (MAI).

Throughout this work, the small set of Kasami sequences [10], considered as pseudo-random (PN) sequences, is used. Kasami sequences have better cross-correlation values than those obtained with other pseudo-random sequences, such as m-sequences [11] or Gold sequences [12]. Complementary codes, like Golay or complementary set of sequences (CSS) sequences, have also been discarded. These codes have the complementary property, thus providing interference cancellation by the addition of the auto-correlation functions from a set of sequences. However, to take advantage of this particular feature, it is necessary to increase the number of emissions, compared to the Kasami case. Moreover, the complementary property severely degrades in an attenuating channel [13].

The small set of Kasami sequences is composed by *K* = 2*^X^*^/2^ sequences of length *L* = 2*^X^* − 1, where *X* must be even. If the length, *L*, of the sequences increases, the number of pseudo-random codes available also increases. The AC and CC sidelobes are limited to $\left\{-1,-2^{\frac{X}{2}}-1,2^{\frac{X}{2}}-1\right\}$. Furthermore, it is possible to use these sequences for asynchronous proposals. In that case, AC and CC levels are not bounded to a fixed range, and they strongly depend on how the sequences are generated. Therefore, interference values in AC and CC aperiodic functions could be worse or better than in the periodic case. The generation of Kasami sequences used in this work is based on the algorithm presented in [14], which involves the selection of those sequences with lower crosstalk values in an asynchronous detection.

**Figure 1.**
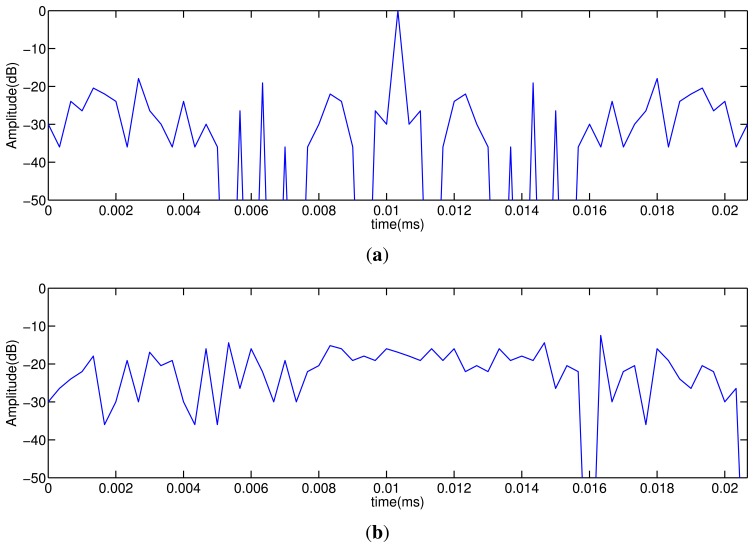
(**a**) Auto-correlation (AC) and (**b**) cross-correlation (CC) functions of a Kasami sequence with *L* = 63 bits modulated in BPSK (binary phase shift keying) using a single carrier period per bit.

An important issue of the coded emission is to adapt the codes to the working frequency of the transducer, ensuring that most of the energy of the emitted code is inside the transducer bandwidth. Due to their easy implementation and their capacity to keep a narrow bandwidth, centered at the carrier frequency, a BPSK (binary phase-shift keying) modulation is used. Figure 1 shows the auto-correlation and cross-correlation functions obtained with Kasami sequences of *L* = 63 bits; the sequences are BPSK modulated, by using a single carrier period per bit. The AC function presents a main lobe that can be clearly identified from the AC and CC interference levels, which are around 20 dB lower. These particular features make Kasami codes suitable for simultaneous emission and reception.

### 2.1. Encoded Phased Array Sectors (ePA_sec_)

**Figure 2.**
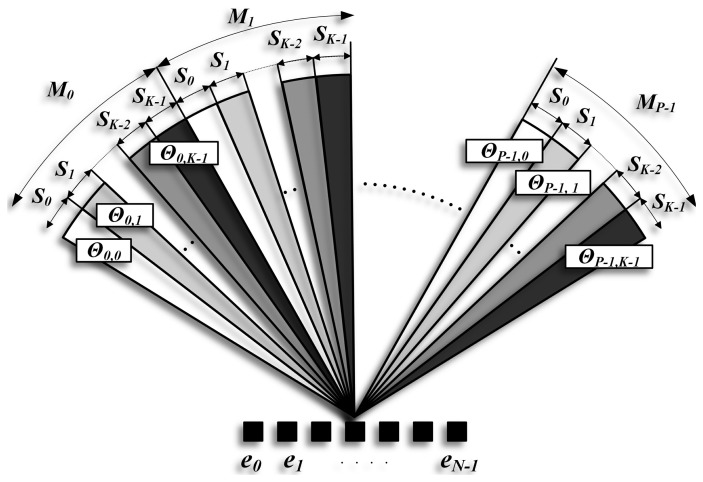
ePA_sec_ (encoded phased array sectors). The scan area is divided into *P* sectors *M_j_*_=0,1,…,_*_P_*_−1_, each one explored with *K* sequences *S_i_*_=0,1,…,_*_K_*_−1_, emitted in different directions Θ*_j_*_,_*_i_*_=0,1,…,_*_K_*_−1_.

The proposed system is based on an array with *N* elements, (*n* = 0,1,…, *N* − 1), which emit encoded signals to scan the medium in only one emission. Figure 2 shows the encoded [ePA_sec_ (encoded phased array sectors)] scheme, where the scan area is divided into *P* sectors (*M_j_*_=0,1,…,_*_P_*_−1_); all of them scanned simultaneously by emitting the same set of sequences, *S_i_*_=0,1,…,_*_K_*_−1_. In every sector, ***j***, each one of the sequences, *i*, is steered in a particular direction (Θ*_j_*_,_*_i_*).

**Figure 3.**
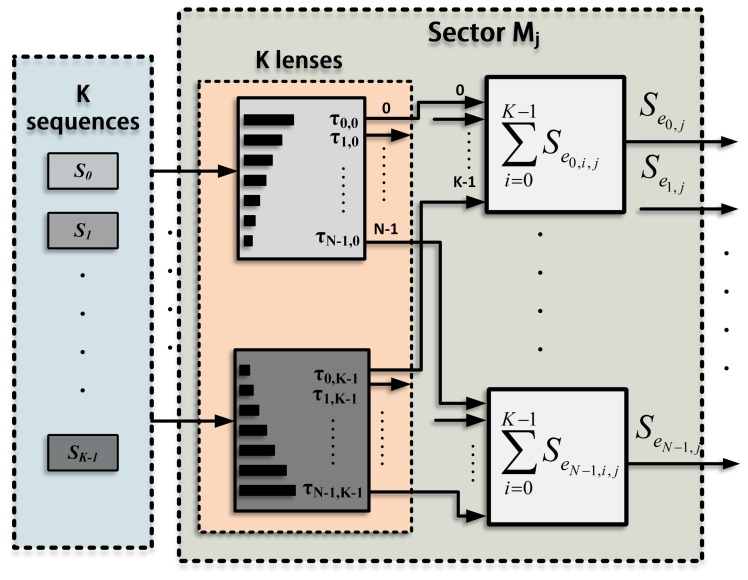
Detail of the emission process in a sector, *M_j_*. The sector is explored by emitting *K* sequences (*S_i_*_=0,1,…,_*_K_*_−1_), where a different lens is applied to each sequence to scan *K* directions. *K* signals *S*_*e*_*n*=0,1,…,*N*−1;*j*__, one per element, are obtained for every sector.

**Figure 4.**
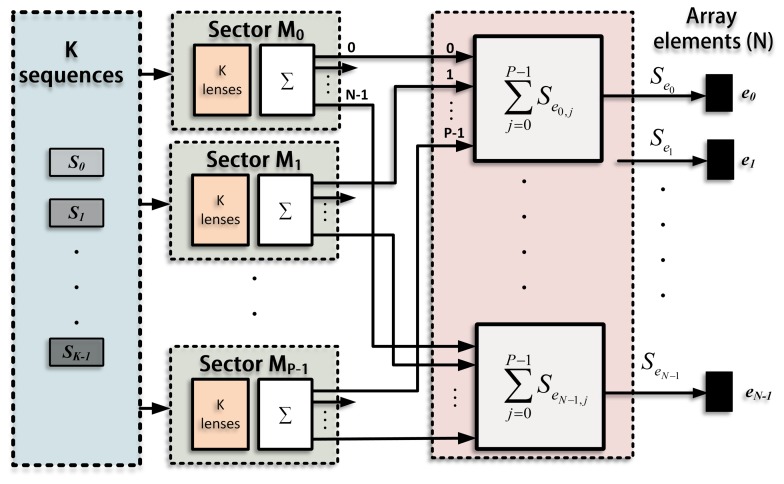
The ePA_sec_ emission stage, where the image is divided into *P* sectors (*M_j_*_=0,1,…,_*_P_*_−1_), each one explored by emitting *K* sequences (*S_i_*_=0,1,…,_*_K_*_−1_). The signals from every sector (*S*_*e*_*n*=0,1,…,*N*−1;*j*=0,1,…,*P*−1__) are added to obtain the final signal to be transmitted by each element (*S*_*e*_*n*=0,1,…,*N*−1__).

The emission process for a single sector, *M_j_*, is shown in Figure 3. A suitable lens, with a different delay for every element (*τ*_*e*_*n*=0,1,…,*N*−1;*j;i*=0,1,…,*K*−1__), is applied to each sequence in order to generate *K* different beams focused along the directions defined in this sector (Θ*_j_*_;i=0,1,…,_*_K_*_−1_). The signals that correspond to the same element are added, thus obtaining *N* excitation signals (*S*_*e*_*n*=0,1,…,*N*−1;*j*__), one per element. The whole emission stage , including all the sectors, is shown in Figure 4, where each sector presents the scheme in Figure 3. The signals from all the sectors are added to obtain the final excitation for each element. Therefore, the excitation applied to an element, *n*, can be defined as the sum of the signals that will drive that element to generate the beams focused along the *K* · *P* directions.

(1) $$\begin{array}{cc} S_{e_{n}}=\sum_{j=0}^{P-1}\sum_{i=0}^{K-1}S_{i}(t-\tau_{e_{n,j,i}}), & n=0,1,\ldots ,N-1 \end{array}$$

**Figure 5.**
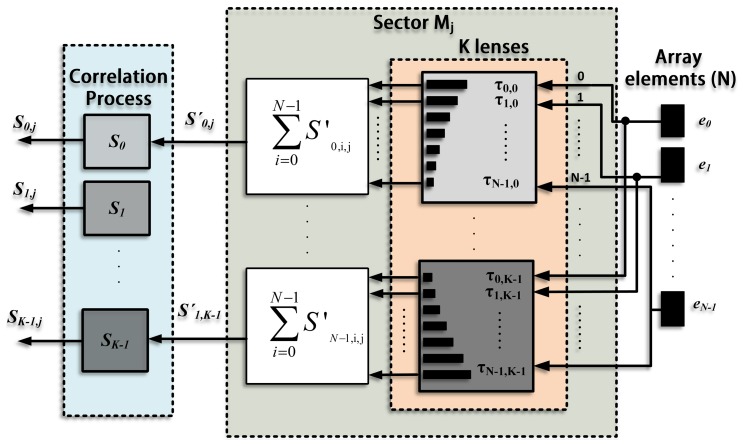
Detail of the acquisition process in one sector, *M_j_*, *K* different lens *τ* are applied. After delaying and summing, *K* signals ( $S_{i=0,1,\ldots ,K-1;j}^{\prime }$) are obtained, each one linked to one of the *K* sequences emitted. Finally, the correlation process is carried out to recover the information.

Figure 5 shows the reception stage in one sector, where *K* different lenses (*τ_n_*_=0,1,…,_*_N_*_−1;_*_i_*_=0,1,…,_*_K_*_−1_) are applied again for every sector. After delaying and adding, *K* signals per sector are obtained ( $S_{i=0,1,\ldots ,K-1;j=0,1,\ldots ,P-1}^{\prime }$), each one linked to one of the *K* sequences emitted.

To recover the information from the medium, a correlation process between the signal, $S_{i,j}^{\prime }$, and its original sequence, *K_i_* , is carried out. Finally, an envelope detector is applied to obtain the lines of the final image. Therefore, the number of image lines simultaneously obtained is *N_L_* = *K* · *P*.

The main advantage of the ePA_sec_ technique is that it allows one to reach the theoretical maximum frame rate, obtaining the full image in only one emission. Hence, the time required to capture data is independent of the number of lines to be acquired.

The main drawback of using coded signals is the crosstalk interference between simultaneously transmitted codes, as was shown in Figure 1. This interference is in the range of −15 dB. Currently, the imaging systems have a wide dynamic range (at least 45 dB) [13], and thus, this interference level could be unacceptable, inserting background noise that degrades the image quality.

### 2.2. Encoded Phased Array and Merging (ePA_merge_)

In order to decrease the crosstalk interference down to an acceptable bound for wide-dynamic range systems, other approaches have to be explored. In this sense, an improvement of the previous technique is presented. The proposal [ePA_merge_ (encoded phased array and merging)] is based on merging several images of the scanned area in order to reduce the influence of the interference on the image quality. It must be remarked that this proposal does not imply a new way to capture data, but an approach to enhance the final result, starting from a set of images obtained with the previous ePA_sec_ technique.

**Figure 6.**
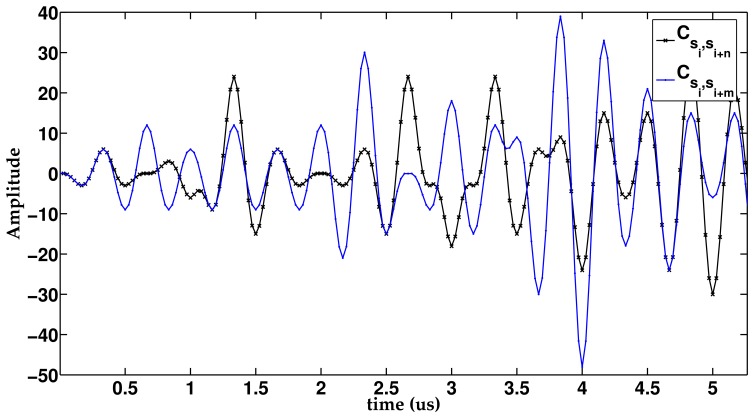
Cross-correlation (CC) function between two pairs of Kasami sequences, (*S_i_*, *S_i_*_+_*_n_*) and (*S_i_*, *S_i_*_+_*_m_*), BPSK modulated with a single carrier period per bit.

The interference between different pairs of sequences of the same set is not equal. To illustrate this idea, Figure 6 shows the CC function between two pairs of Kasami sequences from the same set, (*S_i_*, *S_i_*_+_*_n_*) and (*S_i_*, *S_i_*_+_*_m_*). For both pairs, CC functions are in the same range, but have different values. Taking this into account, it is possible to acquire various images, swapping the direction in which the sequences are emitted each time, in such a manner that the interference at the same steering direction is different in every image. Therefore, the merge softens interference values, because of its variable response. However, the signals coming from scatterers, which have the same features in all the images, appear more intense after merging. As a consequence, the interference reduces its influence in comparison with those echoes coming from the scatterers, thus increasing the contrast resolution.

In the ePA_sec_ proposal, a set of *K* sequences is emitted along each sector, always keeping the same sequence assignment, as was shown in Figure 2. In ePA_merge_, several images are obtained, each one swapping the assignment of sequences. The criterion to swap sequences is that the same sequence should never be used to explore the same line.

The number of raw images depends on the number of sequences. Therefore, from *K* sequences, it is possible to obtain *K* raw images to be merged. So, ePA_merge_ requires *K* emissions to acquire the data. This means that the frame rate achieved by this proposal is lower than that by the ePA_sec_ technique, which needs only one emission. However, ePA_merge_ still attains an acquisition rate higher than that achieved by conventional PA systems, where the number of emissions is equal to the number of image lines.

### 2.3. Selection of the System Parameters

One of the decisive parameters in the ePA technique is the length, *L*, of the PN sequences emitted. As previously discussed, the length, *L*, of the emitted sequences determines the number of sequences available in one set with suitable CC properties. In the ePA_sec_ proposal, the number of lines that can be simultaneously acquired after one emission depends on the sequences available. Moreover, the level of the sidelobes in PN sequences is a function of the code length, *L* [13]. Hence, it would be more advantageous to transmit codes as long as possible. Nevertheless, the code length, *L*, is constrained by the so-called blind zone, *Z_B_*. In the ePA proposal, the same array elements are used to emit and receive, so it is necessary to wait until the whole code is transmitted before starting to acquire data. This reception time-out defines a blind zone or minimum depth penetration for the array. In conventional PA imaging systems, in which short Gaussian pulses are emitted, the blind zone restriction is not so remarkable, since the emission time is negligible in comparison with the round-trip time of the signal. However, emitting coded signals, whose length is far larger, the emission time is not negligible anymore, and the blind zone should be considered.

The emission time depends on the length of the emitted sequences, *L*, the number of carrier cycles per bit in the BPSK modulation, *N_c_*, and the frequency of emission, *f*, if *c* is the propagation speed in the medium. Therefore, the limit range of the blind zone can be expressed as:

(2) $$Z_{B}=\frac{t_{\text{emission}}\cdot c}{2}=\frac{L\cdot N_{c}\cdot c}{2\cdot f}$$

In PA imaging systems, the area to be scanned is mostly located in the near field region, *Z* [15], whose limit can be defined as:

(3) $$Z=\frac{D^{2}}{4\cdot \lambda }=\frac{N^{2}\cdot d^{2}\cdot f}{4\cdot c}$$

where *d* is the pitch of the array and *N* is the number of elements of the array.

It is interesting to study the near field region covered by the blind zone depending on the code length, *L*. Taking a pitch of *d* = λ/2, to avoid grating lobes, the relationship between *Z_B_* and *Z* can be written as:

(4) $$\frac{Z_{B}}{Z}=\frac{8\cdot L\cdot N_{c}}{N^{2}}$$

Table 1 shows the ratio between the near field and blind zone for different array sizes and code lengths, *L*. The values of the table have been obtained for an emission frequency *f* = 3 MHz. The results show that those codes longer than 255 bits are not suitable to scan the near field region. Furthermore, long sequences involve higher emission times, and consequently, the frame rate drops. Arrays with *N* = 64 and *N* = 96 elements emitting sequences of *L* = 63 bits are the best choice to scan distances between 5five and 30 centimeters, which is the range considered in this work.

## 3. Results and Discussion

Different simulations have been performed to analyze the advantages and drawbacks of the ePA_sec_ and ePA_merge_ techniques in comparison with the conventional PA technique.

Arrays with 64 and 96 elements, a pitch of half wavelength and a frequency of 3 MHz are considered in all the cases. It has been assumed that punctual elements have an omnidirectional directivity and a flat frequency response.

A Gaussian envelope pulse with 80% of relative bandwidth has been considered for conventional PA images. In the encoded proposal, the excitation signals are Kasami sequences with *L* = 63 and *L* = 255 bits. The sequences are BPSK modulated by using a single carrier period per bit.

### 3.1. Spatial Resolution

In order to compare the axial and lateral resolution achieved with the ePA and the conventional PA techniques, some images with two punctual reflectors have been obtained in simulation.

The spatial resolution has been quantified using the lateral dynamic range (LDR) and by the axial dynamic range (ADR). For the lateral resolution, two reflectors have been placed at the same depth, *r*, but at different angles, *θ*_1_ and *θ*_2_. The LDR considers the ratio between the received signal from each one of the reflectors, (*r*, *θ*_1_) and (*r*, *θ*_2_), and the signal received from the intermediate angular position (*r*, $\frac{\theta_{1}+\theta_{2}}{2})$. This ratio is expressed in decibels and it allows us to define the minimum angular distance necessary to obtain a specific LDR threshold value.

Table 2 shows the angular distance between two identical punctual reflectors to obtain different LDR values with arrays of 64 and 96 elements. A sweeping in the angular distance, Δ*θ* = *θ*_1_ − *θ*_2_, is performed, in order to search for those angular distances that correspond to different LDR threshold values.

The angular distances required to discriminate two reflectors with LDR values of − 6 dB, −12 dB and −20 dB are very similar in both the ePA_sec_ technique and the PA technique. As can be concluded from the results in Table 2, the ePA_sec_ proposal achieves the same lateral resolution as the conventional PA.

For the axial resolution, two reflectors are placed in the same angle, *θ*, but at different distances from the array, *r*_1_ and *r*_2_. The ADR represents the ratio between the signal received from the reflectors and the signal received from the intermediate range ( $\frac{r_{1}+r_{2}}{2}$). As in the lateral case, it is possible to determine the minimum axial distance that ensures a specific dynamic range threshold value. Table 3 shows the ADR values obtained with PA and ePA_sec_ for arrays with 64 and 96 elements. In this case, a different axial spacing *Δr* = *r*2 − *r*1 between reflectors has been studied. The distance between reflectors can be expressed as a wavelength of the emitted signal. In conventional PA, the value of ADR decreases as the distance between reflectors becomes larger; hence, both reflectors can be distinguished more clearly. On the other hand, in the ePA_sec_ proposal, the ADR scarcely varies with *Δr*, since some inherent interference of the encoding techniques add a background noise, and the ADR level of the conventional PA case cannot be reached. However, for the minimal axial distance, the PA and the ePA_sec_ approaches obtain close ADR values, so it can be concluded that in the coded proposal, the axial resolution barely changes.

### 3.2. Contrast Resolution

In the literature, there are different criteria to measure contrast in imaging; it is sometimes defined as the smallest difference in acoustic impedance that can be displayed, and in other cases, it is referred to as the dynamic range expressed in decibels [16].

In this work, a new parameter, *Q*, has been defined, which provides a more accurate idea of the signal level through the whole image, compared to a reference. This parameter is expressed in decibels and relates the average energy of the pixels in the image under test and its value in the reference image. Mathematically, it can be expressed as:

(5) $$\text{Q}=10\log\left(\frac{\frac{1}{N_{p_{I}}}\sum_{i=0}^{N_{p_{I}}-1}\left|\frac{S_{I_{i}}}{\text{max}(S_{I_{i}})}\right|}{\frac{1}{N_{p_{R}}}\sum_{i=0}^{N_{p_{R}}-1}\left|\frac{S_{R_{i}}}{\text{max}(S_{I_{i}})}\right|}\right)$$

where *N_p_I__* and *N_p_R__* are the number of points in the evaluated image and in the reference image, respectively, and *S_I_i__* and *S_R_i__* are the signals coming from each point in the image to be evaluated and in the reference image. The closer the *Q* parameter is to unity, the closer energy dispersion is in both images.

Table 4 shows the values of *Q* for different images in the ePA techniques, using the PA image as reference. The values have been obtained by scanning a sector of *β* = 60° and by acquiring enough image lines to meet with the Nyquist criterion: *N_l_* = 64 lines for an array of 64 elements and *N_l_* = 104 lines for an array of 96 elements. Since *K* = 8 sequences are emitted, merging is performed from eight ePA_sec_ images.

Both ePA proposals present a loss of contrast, due to the interference between the emitted sequences. ePA_sec_ obtains *Q* values in the range of 5–6 dB, whereas in ePA_merge_, the values achieved are in the range of 1.15–1.5 dB. Merging implies a reduction of 4 dB, thus providing a *Q* value closer to unity.

**Figure 7.**
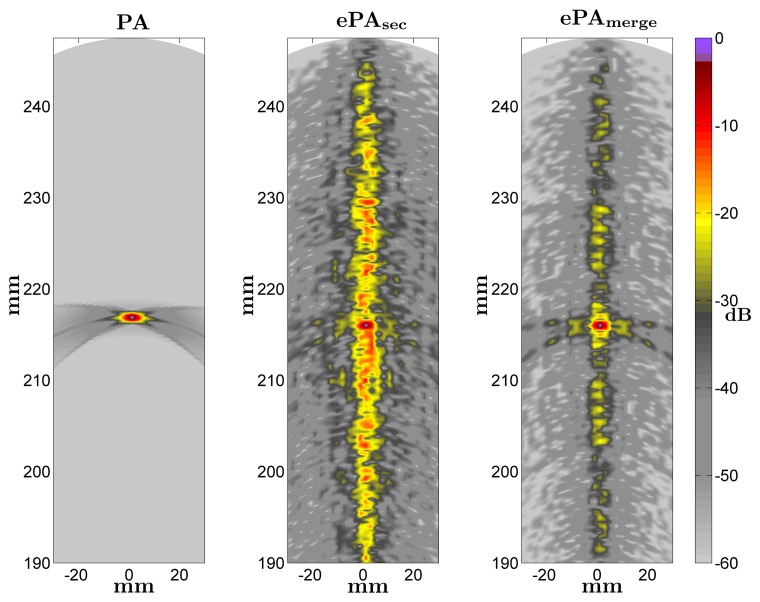
Images obtained with the PA and the proposed ePA techniques. A punctual reflector is located at *r* = 216 mm and *θ* = 0°. An array with *N* = 96 elements and a Kasami sequences of *L* = 63 bits are considered in ePA images.

Figure 7 shows some images obtained with the conventional PA technique and the ePA proposals when a medium with a punctual reflector located at *r* = 216 mm and *θ* = 0° is scanned. An array with *N* = 96 elements and Kasami sequences of *L* = 63 bits are considered in the ePA images. In ePA_sec_, the interference is around −10 dB under the reflector response, whereas ePA_merge_ reduces the background interference level up to −30 dB. Therefore, in both ePA cases, the contrast resolution and the image quality decrease compared to the conventional PA. The interference levels are higher in the direction corresponding to the reflector position, because of the AC interference values.

### 3.3. Temporal Resolution

The main advantage of the ePA technique is that the number of emissions required to scan the whole area is minimum. ePA_sec_ reaches the theoretical maximum frame rate, obtaining the whole information in only one firing. In the ePA_merge_ approach, the final image is formed after merging *K* images obtained with ePA_sec_, where *K* is the number of sequences available. Thus, the number of emissions needed in this case is equal to *K*.

Table 5 shows the acquisition rate, in images per second, achieved by the PA and ePA techniques. To figure out the acquisition rate, only the emission time, the propagation time and the number of emissions have been taken into account. At the reception stage, beamforming, the correlation for the ePA techniques and envelope detection are carried out, so the achieved rates should vary depending on how this process is performed. In all the cases, it has been considered as an angular sector of *β* = 90°, a maximum range depth of *R_max_* = 20 cm and different array sizes of *N* = 96 and *N* = 64 elements. The Kasami sequences with *L* = 63 bits are emitted in ePA proposals.

In the conventional PA, the acquisition rate strongly depends on the number of lines to be acquired, decreasing from 37 to 25 images per second, whether the number of lines grows from 100 to 150. ePA_sec_ achieves 3476 images per second, attaining 93× and 139× speed-up with respect to the PA for *N* = 64 and *N* = 96, respectively. ePA_merge_ achieves 434 images per second, attaining 11× and 17× speed-up with respect to the PA for *N* = 64 and *N* = 96, respectively.

## 4. Conclusions

A new excitation scheme based on introducing encoding techniques in PA imaging systems has been presented. The proposal reduces the acquisition time with respect to the conventional PA technique. The technique consists of assigning a different pseudo-random Kasami sequence to every steering direction. Taking advantage of the suitable cross-correlation properties of the emitted codes, several scan lines can be simultaneously acquired in only one emission.

Simulation results have shown that the encoded proposal scarcely affects the spatial resolution. Theoretically, the inherent interference of Kasami sequences adds a background noise, thus influencing the image contrast, but it has been verified that the ePA_merge_ approach allows one to reduce that noise.

The ePA proposals strongly decrease the emissions required to capture data, making the acquisition process much faster than in conventional PA systems. Thus, ePA_sec_ and ePA_merge_ attain 139× and 17× speed-up with respect to the PA. This feature makes ePA schemes a promising alternative to reduce acquisition time in 2D PA systems.

## Figures and Tables

**Table 1.** The percentage of the near field region, *Z*, occupied by the blind zone, *Z_B_*. Kasami sequences with different lengths, *L*, are considered for an emission frequency *f_c_* = 3 MHz, a propagation speed of *c* = 1500 m/s and different array sizes, *N*. The sequences are BPSK modulated by using a single carrier period per bit, *N_c_* = 1.

		***L*** **= 63**	***L*** **= 255**	***L*** **= 1023**

		***K*** **= 8**	***K*** **= 16**	***K*** **= 32**
***N***	***Z*(cm)**		***Z****_B_***/*Z*(%)**	
**32**	3.2	50	>100	>100
**64**	12.8	12	50	>100
**96**	28.8	5	22	88
**128**	51.2	3	12	50

**Table 2.** Lateral resolution obtained with PA and ePA_sec_ for arrays with 64 and 96 elements, in order to achieve lateral dynamic range (LDR) values of − 6 dB, −12 dB and − 20 dB.

		**R_L_(°)**		
**LDR**		**−6 dB**	**−12 dB**	**−20 dB**
**PA**	***N*** **= 64**	2.35	3.17	5.14
	***N*** **= 96**	1.56	2.11	3.60
**ePA_sec_**	***N*** **= 64**	2.72	4.1	6.2
	***N*** **= 96**	1.85	2.5	4

**Table 3.** Axial dynamic range (ADR) values obtained with PA and ePA_sec_ for array sizes of *N* = 64 and *N* = 96 elements and different axial distances between reflectors (*Δr*). Axial distances are expressed as a wavelength of the signal emitted.

		**ADR(dB)**		
***Δ*r**		**2·λ**	**3·λ**	**10·λ**
**PA**	***N*** **= 64**	−24.51	−61.85	−84.78
	***N*** **= 96**	−24.59	−62.02	−84.86
**ePA_sec_**	***N*** **= 64**	−17.34	−18.53	−20.07
	***N*** **= 96**	−21.49	−17.49	−25.98

**Table 4.** Contrast resolution, *Q*, obtained with the ePA_sec_ and the ePA_merge_ techniques, by scanning a sector *β* = 60° with arrays with 64 and 96 elements. In all the cases, the PA image is used as a reference.

	***N***	64	96

	***N****_l_*	64	104
***Q*** **(dB)**	**ePA_sec_**	5.11	6.08

	**ePAmerge**	1.18	1.53

**Table 5.** Acquisition time for an angular sector *β* = 90° and a maximum range depth *R*_max_ = 20 cm, obtained with the PA and the ePA for array sizes of *N* = 64 and *N* = 96 elements.

	***N***	64	96

	***N*_1_**	100	150
**Acquisition rate (Imag./Sec)**	**PA**	37	25

	**ePA_sec_**	3476	3476

	**ePAmerge**	434	434
